# Supplementary material for: recount3: summaries and queries for large-scale RNA-seq expression and splicing
Source: Genome Biol. 2021 Nov 29;22:323. doi: 10.1186/s13059-021-02533-6 (PMC8628444; doi:10.1186/s13059-021-02533-6)
Supplement: Supplementary file 1 — Additional file 1 Review history. [file 13059_2021_2533_MOESM1_ESM.docx]

Review History

**First round of review**

**Reviewer 1**

**Were you able to assess all statistics in the manuscript, including the appropriateness of statistical tests used?**

Yes: Tests used appeared to be appropriate, however, I did not examine this in depth.

**Comments to author:**

Wilks and colleagues describe an update to their recount resource, recount3. A major, though often unacknowledged, problem in genomics is that reusing public data can be extraordinarily time consuming and unnecessarily difficult. Some publications don't include supplemental tables of gene hit counts for RNA-seq datasets. Other times, researchers may want to rerun an analysis using a different genome annotation. This means researchers have to find and download raw data, realign these data to the genome, rerun quantification or splicing programs, etc. This is a huge computational redundancy and wastes both researcher time and money (compute time). Recount3 helps address this problem by providing a systematic, internally consistent resource of RNA-seq data evaluated both in processed form (gene / isoform / exon counts) and in a semi-raw form (bigwig files of genome coverage). As a PI who often asks students to download data from NCBI GEO and realign it (because there's no bam / bigwig / bedGraph file available), I am excited to learn of this resource of processed files for tens of thousands of studies. I only have a few comments and suggestions:

1) My enthusiasm is high, however I was slightly disappointed when I visited the recount3 website and realized that many biologists will have difficulty accessing the data. The study explorer is helpful, but I could not download bigwig files from the explorer. That would be extremely useful and this resource would be much better if that was possible. It would also help if the resource made it easy for researchers to view bigwig files on the UCSC genome browser.

2) Through study explorer, I browsed one study's data and downloaded the tab-delimited expression values and the metadata files. This was a large study with dozens of SR samples. The metadata were not helpful and I couldn't tell what biological sample corresponded to each SR number. This is not a problem I expect the authors to solve, but is worth noting that sorting out such problems takes considerable work and skill.

3) It would also be helpful if the file extension were "tab" or "txt" for the expression data files and "txt" for the metadata files. ".MD" extensions are interpreted as markdown and launch Xcode on Apple computers by default. That's annoying.

4) The http://rna.recount.bio/docs/index.html website has some instructions on how to use the recount3 resource. Larger vignettes and examples would be helpful, as would directly pointing users to the bioconductor page and the manual at bioconductor (https://bioconductor.org/packages/release/bioc/manuals/recount3/man/recount3.pdf).

5) In the docs website, under "How to get help", all the links to bioconductor help (e.g. https://support.bioconductor.org/?tag=recount3) are dead links. Please check the website for other dead links and repair them.

Minor typo:

Pg 4, line 47 - "where uses can run R", "users".

**Reviewer 2**

**Were you able to assess all statistics in the manuscript, including the appropriateness of statistical tests used?**

No

**Comments to author:**

The paper describes a very useful resource with a wide range of applications. Recount3 is an extension of the already very successful predecessor projects recount and recount2. Now a vast number (> 750.000) of available RNAseq data sets have been uniformly processed with a substantial investment in computing power to build a comprehensive resource which summarizes various aspects of sequencing data for the analysis of gene expression and splicing. The resource is made available for easy access and querying via respective Bioconductor packages, documentation and web pages. The workflow to process the datasets in a uniform way is also made available as a snakemake script, which enables to process new custom data sets and to compare the new data to available data and to analyse it in the context of published data.

Thus, recount3 includes a number of new and useful features. The paper is written clear and concise and it is easy to follow. Title and abstract are appropriate, previous and related work is discussed and referenced via an adequate bibliography. The paper contains two Tables and five Figures as well as an extensive supplement. While the paper does not introduce innovative novel methods the resource will be a very useful and I expect a highly cited resource as basis for liklely very many subsequent studies doen in the bioinformatics community. The current paper describes the tools, computational efforts and quite interesting statistics about the compilation of the recount3 resource. Therefore, I recommend accepting the paper.

Some minor points could be improved though:

The figures, in particular the overviews (e.g. Figure 1) could be more illustrative and more transparent.

The legends of both Tables and Figures should be improved, such that all abbreviations are explained and the Tables and Figures are self-contained. E.g. the first Table 1 has no real legend, just two * and ** footnotes including a new abbrev "jxs". As mentioned above the Tables could be compiled with a little more care:

"# Junctions" => "# Junctions (M)"

"0.037 M" => "0.037"

"440.1 (396**) M" => "440.1 (396**)"

"3.022 M" => "3.022"

While it is mentioned that the numbers in Table 1 and 2 are not the same, this could probably be resolved. On the other hand, the small consistencies are not really important, as the numbers are impressive anyway.

"This table may also includes runs" => "This table may also include runs"

Legends should in general be checked and improved (sometimes sentences are not very clear and concise):

Figure 3 : … "The column mean shows that the average percent of annotated exon-exon junctions decreases as the X-axis increases"

Supplementary Table S4: almost no legend: What is meant with "Terabases"?

Figure 5:

(a) no color differences are visible! (what is the message here?)

(b) hard to distinguish the ~40 different colors (what is the message here?)

(c) LCL (new abbrev!) and blood can be identified (what is the message here?)

Figure S7: similar to Figure 5, but more abbrev, legends and axis labels need explanation.

**Reviewer 3**

**Were you able to assess all statistics in the manuscript, including the appropriateness of statistical tests used?**

Yes

**Comments to author:**

In this manuscript, Wilks and colleagues are describing recount3, a massive update of the popular recount resource in terms of the amount of initial raw data, variety of processed data, and tools for data retrieval and analysis. The work is presented with clarity and in great detail. The organization of the webserver and the documentation are excellent. The analyses presented in the manuscript highlight how recount3 can be an invaluable resource to the broader biology and biomedical community.

The only suggestion I have is an addition to Supplementary Note S1 "Detailed related work".

This note currently mentions existing RNA-seq resources along with the quantification pipeline and the number of accessions available in each. The authors also note that most of them only present gene- and transcript-level summaries, and some, in smaller scales, present splicing-related information. To this, it would be valuable to include a discussion of how specific aspects/features of the recount3 pipeline contribute to making it a better resource (in comparison to existing resources) not just in terms of data coverage but also scalability to future data growth, adaptability to changing genome annotations, maintenance of input data quality, and ease of data and metadata retrieval. Some of these aspects are mentioned in a couple of places in the main manuscript (but not in contrast to existing resources) but it would be good to draw up such a comparison in this note. The authors should consider adding refine.bio to this discussion.

**Authors Response**

**Point-by-point responses to the reviewers’ comments:**

Reviewer #1:

Wilks and colleagues describe an update to their recount resource, recount3. A major, though often unacknowledged, problem in genomics is that reusing public data can be extraordinarily time consuming and unnecessarily difficult. Some publications don't include supplemental tables of gene hit counts for RNA-seq datasets. Other times, researchers may want to rerun an analysis using a different genome annotation. This means researchers have to find and download raw data, realign these data to the genome, rerun quantification or splicing programs, etc. This is a huge computational redundancy and wastes both researcher time and money (compute time). Recount3 helps address this problem by providing a systematic, internally consistent resource of RNA-seq data evaluated both in processed form (gene / isoform / exon counts) and in a semi-raw form (bigwig files of genome coverage). As a PI who often asks students to download data from NCBI GEO and realign it (because there's no bam / bigwig / bedGraph file available), I am excited to learn of this resource of processed files for tens of thousands of studies. I only have a few comments and suggestions:

1) My enthusiasm is high, however I was slightly disappointed when I visited the recount3 website and realized that many biologists will have difficulty accessing the data. The study explorer is helpful, but I could not download bigwig files from the explorer. That would be extremely useful and this resource would be much better if that was possible. It would also help if the resource made it easy for researchers to view bigwig files on the UCSC genome browser.

*We agree with the reviewer that bigWig files should be easier to download and use. We followed the reviewer's two suggestions here, extending the Study Explorer to present the links to the bigWig files associated with the selected study. While this can require a lot of "real estate" in the user interface, we mitigated this by including a download button for the “Base-pair coverage bigWig file URLs” through which you can download a CSV file with the URLs for the bigWig files.*

*Further, as suggested, we added a link to the Study Explorer that allows the user to easily open bigWig files in the UCSC genome browser. An example link for mouse project DRP001299 is https://genome.ucsc.edu/cgi-bin/hgTracks?db=mm10&lastVirtModeType=default&lastVirtModeExtraState=&virtModeType=default&virtMode=0&nonVirtPosition=&position=chr1&hubUrl=https:// raw.githubusercontent.com/LieberInstitute/recount3-docs/master/UCSC_hubs/mouse/sra_DRP0 01299/hub.txt. To provide this, we added the create_hub() function to the recount3 R/Bioconductor package and used it at https://github.com/LieberInstitute/recount3-docs/blob/master/UCSC_hubs/create_hubs.R. This new R function creates the UCSC track hub configuration files used in the above UCSC Genome Browser link. For example, for the mouse project DRP001299 that would be https://github.com/LieberInstitute/recount3-docs/tree/master/UCSC_hubs/mouse/sra_DRP0012 99.*

2)	Through study explorer, I browsed one study's data and downloaded the tab-delimited expression values and the metadata files. This was a large study with dozens of SR samples. The metadata were not helpful and I couldn't tell what biological sample corresponded to each SR number. This is not a problem I expect the authors to solve, but is worth noting that sorting out such problems takes considerable work and skill.

*We agree that a typical user will not prefer to interact with the metadata via the raw files that we originally linked to from the Study Explorer. We expect most users to access study data and metadata using the recount3 R/Bioconductor package. To guide users toward this option, we changed the display on the Study Explorer so that the study-specific information that appears below a selected study first lists the commands that can be used to load the study via the recount3 package (see screenshot in response to point #1). When the user executes the commands displayed, the metadata will already be at hand, combined with the data in the form of a SummarizedExperiment (or RangedSummarizedExperiment) object.*

*The links we originally presented are still present, but now below the instructions for how to access the data from the recount3 package. That section has been renamed to "Raw files for this study" to emphasize that it is not the primary or recommended way to access the underlying data and metadata.*

3)	It would also be helpful if the file extension were "tab" or "txt" for the expression data files and "txt" for the metadata files. ".MD" extensions are interpreted as markdown and launch Xcode on Apple computers by default. That's annoying.

*We see the reviewer's point about the file extension used for the metadata. In retrospect, we wish that we had used an extension that was more reflective of the data format.*

*That said, we decided not to make this change as part of this paper revision. This is because it would necessitate a coordinated set of changes to multiple aspects of the service, and would be a "breaking" change in the sense that users who had taken note of the old file extensions would have to update to the new extensions or face non-working URLs. It would also disrupt collaborators and power users who have made private mirrors of the recount3 data. While we are certainly willing to make disruptive changes like this, and such changes are healthy in the long run, we would like to save this particular change for a future time when we are otherwise extending the resource.*

4)	The http://rna.recount.bio/docs/index.html website has some instructions on how to use the recount3 resource. Larger vignettes and examples would be helpful, as would directly pointing users to the bioconductor page and the manual at bioconductor (https://bioconductor.org/packages/release/bioc/manuals/recount3/man/recount3.pdf).

*We agree with the reviewer that the documentation we originally posted could have been better organized and more expansive. At http://rna.recount.bio/docs/quick-access.html and*

*http://rna.recount.bio/docs/bioconductor.html and several other locations, we have now added links to the Bioconductor websites for our packages. We also made edits to further highlight our more extensive vignette documents https://github.com/LieberInstitute/recount3-docs/commit/ac510b59f091c325bfce272645f9f92af8 6949ef. See the changes highlighted in the screenshots below.*

5) In the docs website, under "How to get help", all the links to bioconductor help (e.g. https://support.bioconductor.org/?tag=recount3) are dead links. Please check the website for other dead links and repair them.

*The reviewer is correct, and we have now fixed this at*

*https://github.com/LieberInstitute/recount3-docs/commit/b5ece34b270180bc85aa7cc7eb901df4 09d23b63.*

Minor typo:

Pg 4, line 47 - "where uses can run R", "users".

*Thank you for noticing this typo. We have fixed this.*

Reviewer #2:

Recount3

The paper describes a very useful resource with a wide range of applications. Recount3 is an extension of the already very successful predecessor projects recount and recount2. Now a vast number (> 750.000) of available RNAseq data sets have been uniformly processed with a substantial investment in computing power to build a comprehensive resource which summarizes various aspects of sequencing data for the analysis of gene expression and splicing. The resource is made available for easy access and querying via respective Bioconductor packages, documentation and web pages. The workflow to process the datasets in a uniform way is also made available as a snakemake script, which enables to process new custom data sets and to compare the new data to available data and to analyse it in the context of published data.

Thus, recount3 includes a number of new and useful features. The paper is written clear and concise and it is easy to follow. Title and abstract are appropriate, previous and related work is discussed and referenced via an adequate bibliography. The paper contains two Tables and five Figures as well as an extensive supplement. While the paper does not introduce innovative novel methods the resource will be a very useful and I expect a highly cited resource as basis for liklely very many subsequent studies doen in the bioinformatics community. The current paper describes the tools, computational efforts and quite interesting statistics about the compilation of the recount3 resource. Therefore, I recommend accepting the paper.

Some minor points could be improved though:

The figures, in particular the overviews (e.g. Figure 1) could be more illustrative and more transparent.

*We agree with the reviewer and our responses below lay out specific steps we took to improve the figures.*

The legends of both Tables and Figures should be improved, such that all abbreviations are explained and the Tables and Figures are self-contained. E.g. the first Table 1 has no real legend, just two * and ** footnotes including a new abbrev "jxs". As mentioned above the Tables could be compiled with a little more care:

"# Junctions" => "# Junctions (M)"

"0.037 M" => "0.037"

"440.1 (396**) M" => "440.1 (396**)"

"3.022 M" => "3.022"

*We agree with all of the reviewer's critiques and suggestions regarding Table 1 and its caption. The table now has a more descriptive caption, beginning "Size and computational cost of Monorail runs." The "M" millions modifier was moved to the column header as suggested. Where we previously presented two alternate versions of a tally, we now present just one version and explain it in a footnote. Overall, we think Table 1 is much clearer now.*

While it is mentioned that the numbers in Table 1 and 2 are not the same, this could probably be resolved. On the other hand, the small consistencies are not really important, as the numbers are impressive anyway.

*We agree with the reviewer that we could have done more to reconcile the numbers in the two tables. We added the following text to explain differences between Tables 1 & 2: "These numbers tally the number of run accessions processed, which can exceed the numbers in Table 1 due to some runs being processed multiple times, and due to runs that were later removed for QC or metadata reasons. Missing from this table are several thousand SRA human run accessions that were analyzed on MARCC but whose log files were discarded."*

"This table may also includes runs" => "This table may also include runs"

*We made this change, thank you.*

Legends should in general be checked and improved (sometimes sentences are not very clear and concise):

*We made several editing changes to the captions so as to improve clarity. A few include:*

*l	Clarifications to the caption and the presentation of results in Table 1 (discussed above)*

*l	Clarifications to the caption of Table 2, including details on why some totals do not match between Tables 1 and 2*

*l	Elaboration of acronym in Figure 1 caption*

*l	Clarification in caption of terms used within Figure 3*

*l	More detailed caption of Figure 5 that both defines an acronym (LCL) and better explains axes*

*l	Longer and more detailed caption for Supplementary Table 4*

*l	Detailed figure captions for all new figures (related to our response to the Figure-5 critique below)*

Figure 3 : ... "The column mean shows that the average percent of annotated exon-exon junctions decreases as the X-axis increases"

*We have edited this sentence and it now reads like this:*

*The column mean, Col.Mean, shows that the average percent of annotated exon-exon junctions decreases as the cell type specificity increases on the X-axis.*

Supplementary Table S4: almost no legend: What is meant with "Terabases"?

*We agree with the reviewer that Supplementary Table 4 deserved a more detailed caption, as well as a clearer presentation of the data in the figure. We made two major changes. First, we changed the data so that the most inclusive rows were inclusive only of the run accessions that ultimately made it into the recount3 resource. Previously, there were rows labeled "Pre-scRNA filtered" for human and mouse, and that row was inclusive of all run accessions including many that we eventually filtered out for using non-Smart-seq-based single-cell protocols. In the new table, only run accessions that were ultimately included in recount3 are counted, and so the new rows labeled "Bulk" and "Smart-seq" actually add to the row labeled "Total."*

*Second, we gave the table a more descriptive caption. The caption now reads: "Bulk versus single-cell run accessions processed. Metadata fields were used to subdivide the run accessions into "Smart-seq" and "Bulk" categories, according to criteria described in Supplementary Note S9."*

Figure 5:

(a)	no color differences are visible! (what is the message here?)

(b)	hard to distinguish the ~40 different colors (what is the message here?)

(c)	LCL (new abbrev!) and blood can be identified (what is the message here?)

*We have substantially revised this results section. As part of this revision we now include 4 additional supplemental figures (S8, S9, S10 and S11). We believe the entire narrative and graphical presentation is substantially clearer. What follows is our comments on these specific questions from the reviewer.*

*In (a) there are two messages (1) we want to show the full PCA plot with very little coloring and (2) library size does not drive the first two principal components (confirmed by a correlation analysis). Figure 5a does not really show the grey points - partly because there is no relationship - so the reviewer might want to contrast with Supplementary Figure 11a where there is a relationship. In both cases, the relationship is actually better ascertained by computing a correlation, which is now included in the text.*

*In (b), the main message is that labelled tissue samples cluster together. We appreciate that this is really hard to see in this single plot due to the many different tissues. To help with this, we have included a new supplemental figure where we have faceted on tissue. The reviewer will appreciate that this figure is too big to be a main figure.*

*In (c) the message is that blood -- and blood-related samples which include lymphoblastoid cell lines (LCLs) cluster together and form a rather large group. LCLs is a rather specific - but abundant - cell type / “tissue” in SRA. The first principal component is related to the amount of hemoglobin (new supplemental figure).*

Figure S7: similar to Figure 5, but more abbrev, legends and axis labels need explanation.

*We have modified this figure to spell out the abbreviations and we have re-written the caption. We believe the figure is substantially more clear.*

Reviewer #3:

In this manuscript, Wilks and colleagues are describing recount3, a massive update of the popular recount resource in terms of the amount of initial raw data, variety of processed data, and tools for data retrieval and analysis. The work is presented with clarity and in great detail. The organization of the webserver and the documentation are excellent. The analyses presented in the manuscript highlight how recount3 can be an invaluable resource to the broader biology and biomedical community.

The only suggestion I have is an addition to Supplementary Note S1 "Detailed related work". This note currently mentions existing RNA-seq resources along with the quantification pipeline and the number of accessions available in each. The authors also note that most of them only present gene- and transcript-level summaries, and some, in smaller scales, present splicing-related information. To this, it would be valuable to include a discussion of how specific aspects/features of the recount3 pipeline contribute to making it a better resource (in comparison to existing resources) not just in terms of data coverage but also scalability to future data growth, adaptability to changing genome annotations, maintenance of input data quality, and ease of data and metadata retrieval. Some of these aspects are mentioned in a couple of places in the main manuscript (but not in contrast to existing resources) but it would be good to draw up such a comparison in this note.

*We agree and we greatly expand the discussion at the end of Supplementary Note 1. Specifically, we added a new bulleted set of paragraphs, with the introductory sentence "Some design improvements in recount3 can be more directly compared and contrasted with prior projects." We then went on to describe four key aspects of the recount3 and how recount3 can be compared and contrasted to prior work based on each of these aspects. In short, these four aspects are (a) our use of a grid-based architecture and containerized workflow, which some prior projects have also done, (b) our unique strategy of quantifying genes and exons from bigWig files and how this reduces the barrier to future requantification using new annotations, (c] our uniquely annotation-agnostic analysis pipeline that does not use gene annotation to inform spliced alignment, (d) our inclusion of the Snaptron infrastructure -- which had previously been a separate project -- in recount3, allowing rapid queries.*

The authors should consider adding refine.bio to this discussion.

*We agree that this is a very relevant project and we now refer to and cite it in Supplementary Note S1.*

**Second round of review**

**Reviewer 1**

The revised manuscript is even more useful than the original submission and addressed all of my concerns adequately.
